# Total weight loss rather than preoperative body mass index correlates with remission of irregular menstruation after sleeve gastrectomy in patients with polycystic ovary syndrome

**DOI:** 10.3389/fendo.2024.1355703

**Published:** 2024-03-11

**Authors:** Yian Zhao, Sisi Xiong, Teng Liu, Jiaxin Shu, Tao Zhu, Shumin Li, Mingwei Zhong, Shigang Zhao, Xin Huang, Shaozhuang Liu

**Affiliations:** ^1^ Division of Bariatric and Metabolic Surgery, Department of General Surgery, Qilu Hospital of Shandong University, Jinan, China; ^2^ Center for Reproductive Medicine, Renji Hospital, School of Medicine, Shanghai Jiao Tong University, Shanghai, China; ^3^ Division of Bariatric and Metabolic Surgery, Department of General Surgery, the First Affiliated Hospital of Shandong First Medical University, Jinan, China; ^4^ Center for Reproductive Medicine, Cheeloo College of Medicine, Shandong University, Jinan, China

**Keywords:** obesity, total weight loss, polycystic ovary syndrome, sleeve gastrectomy, irregular menstruation

## Abstract

**Introduction:**

Polycystic ovary syndrome (PCOS) is the most common endocrinopathy affecting reproductive-aged women. Some retrospective studies with small sample sizes have reported that bariatric metabolic surgery is effective in remission of irregular menstruation in patients with PCOS and obesity. However, the correlation between preoperative body mass index (BMI), postoperative weight loss, and remission of irregular menstruation in patients with obesity and PCOS after sleeve gastrectomy (SG) is lack of consensus.

**Methods:**

We enrolled 229 participants with obesity and PCOS who underwent SG. All patients were followed up for one year after surgery. Remission of irregular menstruation was defined as a spontaneous consecutive six-month menstrual cycle in one year. Subgroup analysis was conducted using tertiles of preoperative BMI and postoperative total weight loss (TWL)% to determine their correlation with the remission of irregular menstruation after SG.

**Results:**

79.03% (181/229) patients achieved remission of irregular menstruation one year after SG with a TWL% of 33.25 ± 0.46%. No significant difference was detected in the remission rate among the subgroups with different BMI (*P*=0.908). TWL% was correlated with the remission of irregular menstruation (OR 1.78, 95% CI 1.18-2.69, *P*<0.05).

**Conclusions:**

SG had a significant effect on the remission of irregular menstruation in patients with obesity and PCOS. Preoperative BMI did not emerge as a decisive factor correlated with remission; instead, TWL% showed potential as a key factor.

## 1 Introduction

Polycystic ovary syndrome (PCOS) is the most common endocrinopathy affecting reproductive-aged women with a 10%-13% prevalence (1), accounting for nearly 80% of infertility cases (2). The clinical presentation of PCOS is complex, with reproductive, metabolic, and psychological features that impact across the lifespan (3). Among the typical characteristics of PCOS, irregular menstruation is a key concern for patients because of infertility and an increased risk of endometrial cancer. PCOS is strongly associated with obesity and other metabolic disorders. More than 60% of women with PCOS are estimated to be overweight or obese (4). Obesity further amplifies and worsens all the metabolic and reproductive outcomes of PCOS (5).

Weight management is recommended as the mainstay treatment for PCOS (1), and symptoms commonly improve with 5%-10% weight loss (6). However, when combined with morbid obesity, the required 25%-50% weight loss is difficult to achieve, depending on lifestyle or medical treatment (7). In addition, patients with PCOS and obesity tend to respond poorly to pharmacological treatments for ovarian stimulation and assisted reproductive technology (8). Therefore, the traditional treatment methods for patients with PCOS and morbid obesity have encountered dilemmas.

As the most effective and reliable treatment for morbid obesity, bariatric metabolic surgery (BMS) also results in greater improvement in obesity-related comorbidities, including type 2 diabetes, hypertension, dyslipidemia, sleep apnea, and osteoarthritis, than non-surgical treatment (9). Sleeve gastrectomy (SG) is the most common bariatric metabolic procedure, accounting for more than 60% of both total and primary procedures globally (10). Previous studies with small sample sizes reported that BMS was effective for PCOS in terms of improving ovulatory dysfunction, hyperandrogenism, and infertility (11–13). However, BMS is still conditionally recommended for patients with PCOS and obesity in the 2023 International Assessment and Management of PCOS owing to the absence of high-level evidence (1). Moreover, although a correlation between PCOS and obesity has been confirmed, there is no consensus on whether the degree of preoperative obesity influences PCOS improvement after BMS. Further research is required to elucidate the effect of postoperative weight loss on the prognosis of postoperative PCOS.

To confirm the efficacy of SG in patients with PCOS and obesity, we conducted a prospective multi-center cohort named “Sleeve Gastrectomy for Obese Polycystic Ovary Syndrome” (SGOP). Based on the subdatasets of the SGOP cohort, the current study aimed to investigate the correlation of preoperative BMI and postoperative weight loss with remission of irregular menstruation after SG.

## 2 Materials and methods

### 2.1 Study design and patients

Beginning in January 2020, patients in the SGOP cohort were prospectively recruited at Qilu Hospital of Shandong University (Center A) in Jinan, Shandong province and First Affiliated Hospital of Shandong First Medical University (Center B) in Jinan, Shandong province. Among the SGOP serial studies, the SGOP-01 study aimed to compare the efficacy of SG with that of drug therapy, and was registered in the Chinese Clinical Trial Registry with the identification number ChiCTR1900026845.

A subset of patients from the SGOP-01 study was recruited for the current study. The current study was approved by the Medical Ethics Committee of Qilu Hospital of Shandong University. The inclusion criteria of this study were as follows: (1) BMI≥32.5 kg/m^2^ or BMI≥27.5kg/m^2^ combined with diabetes or two components of metabolic syndrome, (2) aged 18–42 years, and (3) PCOS with irregular menstruation. PCOS was diagnosed based on the 2003 Rotterdam criteria (14). Patients who received pharmacological treatment for PCOS, had previously undergone BMS, had incomplete preoperative data, or had poorly controlled psychological disorders were excluded.

All patients were followed up for at least one year after SG. Remission of irregular menstruation was defined as a spontaneous consecutive six-month menstrual cycle (21-35 days) in one year after SG.

### 2.2 Procedure and follow-up

All SG procedures at each center were performed by the same experienced bariatric and metabolic teams. The surgical process was consistent with that previously described (15). All sleeve gastrectomy at each center was performed laparoscopically by the same experienced bariatric and metabolic teams. Briefly, the greater curvature was dissected free from the momentum starting 2–4 cm from the pylorus and up to the angle of His, with a tubular sleeve created using a 36-Fr bougie. All the patients received suggestions for postoperative diet and exercise. Weight reduction and menstrual status were recorded each month after SG. The total weight loss (TWL)% was calculated to evaluate weight loss.

### 2.3 Biochemical analysis

Sex hormone, thyroid function, blood lipid analysis, and fasting plasma glucose (FPG) levels were measured using a Roche Cobas 8000 modular analyzer system (Roche Diagnostics, IN, USA). Plasma insulin and C-peptide levels were determined using a two-site enzymatic assay with a Tosoh 2000 Autoanalyzer (Tosoh Corp., Tokyo, Japan). Homeostatic model assessment of insulin resistance (HOMA-IR) was performed as follows: fasting insulin (mU/mL) × FPG (mmol/L)/22.5 (16).

### 2.4 Oral glucose tolerance test

After fasting overnight, the participants underwent a 2-h oral glucose tolerance test (OGTT) (75 g of glucose in 250 mL of water). Blood samples were collected in chilled EDTA tubes at 0, 30, 60, and 120 min after glucose intake. The areas under the curve (AUC) of glucose and insulin levels during the OGTT were calculated using the trapezoidal method.

### 2.5 Body composition via dual-energy X-ray absorptiometry

Body composition was determined via DEXA using the HOLOGIC DELPHI system with QDR software, v.11.1 (Hologic, Bedford, MA, USA). Four trained and certified personnel performed whole-body scans. Body fat ratio was calculated as fat volume/body volume, and body lean percentage was calculated as lean mass/body mass.

### 2.6 Statistical analysis

Statistical analyses were performed using IBM SPSS Statistics Version 25.0 for Windows (SPSS Inc, Chicago, IL, USA). Continuous variables are presented as the mean ± SEM, or as the median with the interquartile range. One-way analysis of variance (ANOVA), the Kruskal Wallis H test, t-test and the Mann-Whitney U test were used to compare continuous variables and differences in mean values. The chi-square test was used to compare differences in menstrual outcomes among the groups. The TWL% changes over time among groups were analyzed using two-way ANOVA, followed by the Bonferroni *post hoc* test, and the results were reported as ^A^ *P* by group, ^B^ *P* over time, and ^C^ *P* due to the interaction of the two factors. Univariate logistic regression analysis was performed to assess the correlation between TWL% and remission of irregular menstruation. In all analyses, *P*<0.05 was considered statistically significant.

## 3 Results

### 3.1 Participants

**Figure 1:**
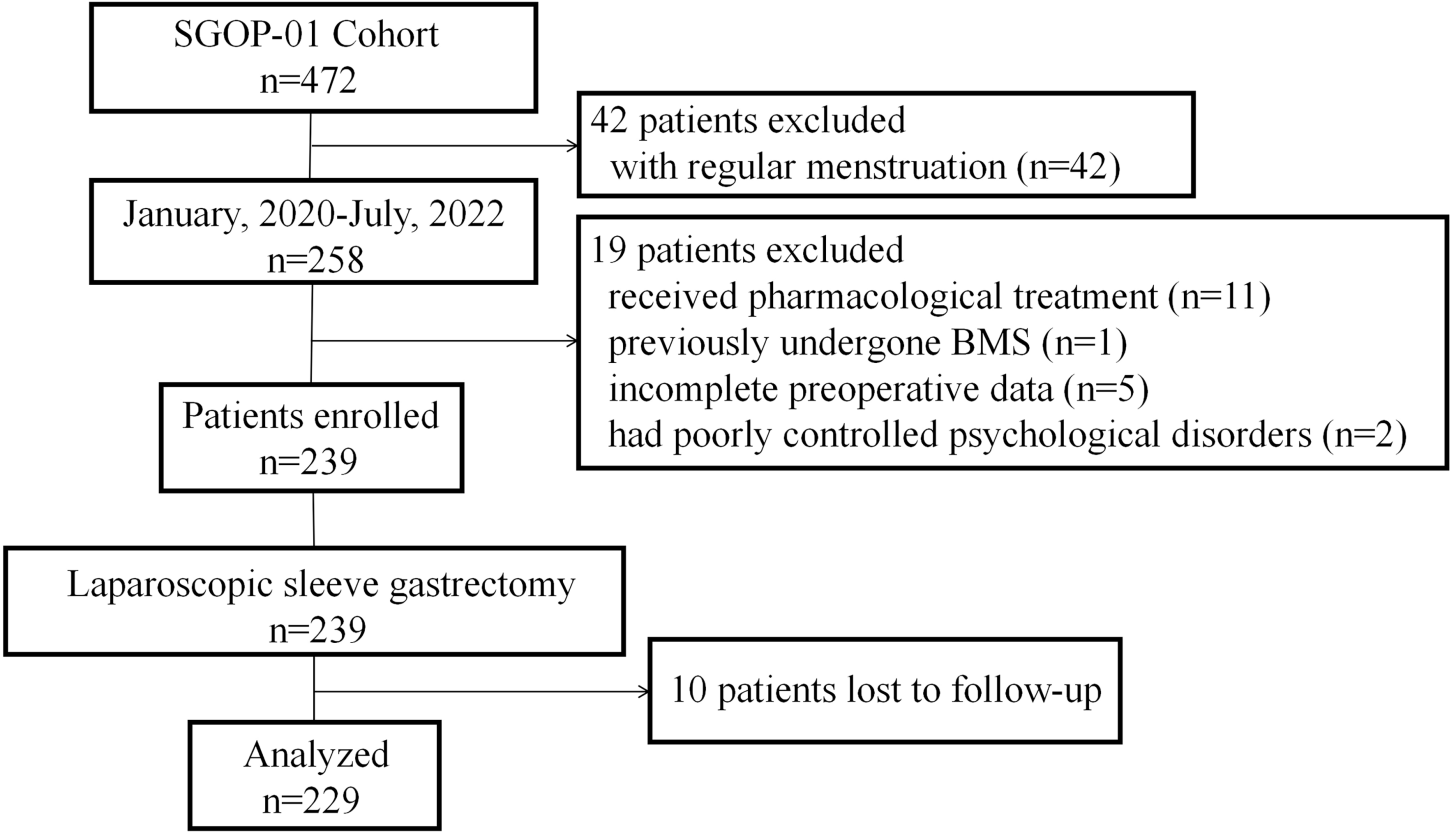
Flowchart of study participants.

Between January 8, 2020, and July 1, 2022, a total of 229 patients (mean [SEM] age, 28.68 [0.35] years; mean [SEM] BMI, 40.91 [0.44] kg/m^2^) with oligo-/anovulatory PCOS and obesity underwent SG in centers A and B (**Figure 1**). All patients completed at least one-year follow-up.

### 3.2 Participants’ baseline characteristics

All participants were divided according to tertiles of baseline BMI (group A, ≤37.65 kg/m^2^; group B, >37.66, ≤42.98 kg/m^2^; group C, >42.99 kg/m^2^), and participants’ characteristics are shown in **Table 1**. The waist-hip ratio (WHR), body fat ratio, and body lean percentage were significantly different among groups A, B, and C (*P*<0.05). No significant differences were detected among the three groups with respect to glycolipid metabolism, thyroid function, or sex hormone levels, except anti-mullerian hormone (AMH) (*P*<0.01). As BMI increased, AMH levels decreased, and there was a significant difference between groups A and C (*P*<0.01).

**Table 1:** Baseline characteristic of participants with obesity and oligo-/anovulatory polycystic ovary syndrome.

Variables	Total Participants (n=229)	Group A (n=77) BMI ≤ 37.65 kg/m^2^	Group B (n=76) BMI ≥ 37.66, ≤ 42.98 kg/m^2^	Group C (n=76) BMI ≥ 42.99 kg/m^2^	*P* Value
Age, years	28.68 ± 0.35	28.88 ± 0.54	28.46 ± 0.67	28.70 ± 0.63	0.89
BMI, kg/m^2^	40.31(35.96, 44.64)	35.05(32.99, 35.96)	40.32(39.05, 41.74)^**^	46.40(44.86, 50.78)^##, &&^	<0.01
WHR	0.94 ± 0.01	0.93 ± 0.01	0.94 ± 0.01	0.96 ± 0.01^#^	0.04
Body fat ratio, %	43.75 ± 0.31	41.54 ± 0.44	43.95 ± 0.54^**^	45.78 ± 0.50^##, &^	<0.01
Body lean percentage, %	51.71 ± 0.33	54.66 ± 0.36	51.81 ± 0.60^**^	48.64 ± 0.50^##, &&^	<0.01
Testosterone, nmol/L	1.76 ± 0.05	1.74 ± 0.09	1.83 ± 0.09	1.69 ± 0.09	0.56
Estradiol, pmol/L	140.00(62.48, 195.10)	123.40(59.34, 174.44)	134.05(62.18, 213.30)	145.60(80.51, 193.35)	0.56
Progesterone, nmol/L	0.42(0.16, 0.77)	0.50(0.19, 1.03)	0.42(0.16, 0.78)	0.38(0.16, 0.70)	0.30
Prolactin, uIU/ml	283.90(213.40, 411.70)	283.90(200.13, 391.35)	265.45(227.51, 457.28)	288.15(203.80, 408.20)	0.53
AMH, ng/ml	4.91 ± 0.19	5.67 ± 0.36	4.97 ± 0.34	4.07 ± 0.25^##^	<0.01
LH, mIU/ml	8.38 ± 0.31	8.58 ± 0.61	8.30 ± 0.56	8.25 ± 0.44	0.90
FSH, mIU/ml	5.08 ± 0.11	4.99 ± 0.19	4.91 ± 0.19	5.34 ± 0.17	0.22
LH/FSH	1.70 ± 0.06	1.81 ± 0.13	1.72 ± 0.10	1.59 ± 0.08	0.38
FT3, pmol/L	5.07 ± 0.07	5.09 ± 0.16	5.09 ± 0.08	5.04 ± 0.09	0.94
FT4, pmol/L	15.26 ± 0.19	15.58 ± 0.40	14.87 ± 0.28	15.32 ± 0.32	0.32
TSH, uIU/ml	2.99 ± 0.44	2.47 ± 0.19	2.68 ± 0.18	3.83 ± 1.29	0.40
FPG, mmol/L	6.43 ± 0.20	6.36 ± 0.36	6.26 ± 0.35	6.66 ± 0.33	0.69
Fasting C-peptide, ng/ml	3.60 ± 0.14	3.45 ± 0.23	3.60 ± 0.28	3.75 ± 0.21	0.69
Fasting insulin, uIU/ml	33.99 ± 2.22	32.84 ± 3.08	38.14 ± 5.46	31.02 ± 2.31	0.40
HbA1_c,_ %	6.49 ± 0.10	6.41 ± 0.20	6.34 ± 0.17	6.73 ± 0.16	0.25
HOMA-IR	6.87(4.63, 11.46)	6.91(4.13, 11.03)	6.70(4.36, 10.62)	7.37(5.34, 11.85)	0.32
AUC_glucose_	21.28 ± 0.55	20.97 ± 0.93	20.88 ± 1.01	22.00 ± 0.90	0.66
AUC_insulin_	231.14 ± 8.13	235.51 ± 15.36	246.02 ± 12.54	211.83 ± 14.07	0.22
Triglyceride, mmol/L	1.59(1.23, 2.33)	1.80(1.31, 2.85)	1.56(1.27, 2.24)	1.53(1.05, 2.00)	0.08
Total cholesterol, mmol/L	4.80 ± 0.06	4.80 ± 0.11	4.73 ± 0.09	4.87 ± 0.11	0.63
HDL-C, mmol/L	1.05(0.94, 1.16)	1.03(0.91, 1.14)	1.05(0.95, 1.17)	1.06(0.95, 1.17)	0.87
Non-HDL-C, mmol/L	3.64(3.16, 4.22)	3.72(3.16, 4.29)	3.59(3.25, 4.01)	3.66(3.13, 4.44)	0.84
NEFA, umol/dL	68.71 ± 1.35	65.10 ± 2.65	68.73 ± 1.88	72.34 ± 2.39	0.09

Data are presented as mean ± SE, or median (25th percentile, 75th percentile). *P < 0.05, **P < 0.01 Group A vs. Group B;#P < 0.05, ##P < 0.01 Group A vs. Group C;&P < 0.05, &&P < 0.01 Group B vs. Group C.

WHR, waist hip ratio; AMH, anti-mullerian hormone; LH, luteinizing hormone; FSH, follicle-stimulating hormone; FT3, free triiodothyronine; FT4, free thyroxine; TSH, thyroid stimulating hormone; FPG, fasting plasma glucose; HbA1c, glycated hemoglobin; HOMA-IR, homeostatic model assessment of insulin resistance; AUC, area under the curve; HDL-C, high density lipoprotein-cholesterol; NEFA, nonesterified fatty acid.

### 3.3 Weight loss effect after SG

**Figure 2:**
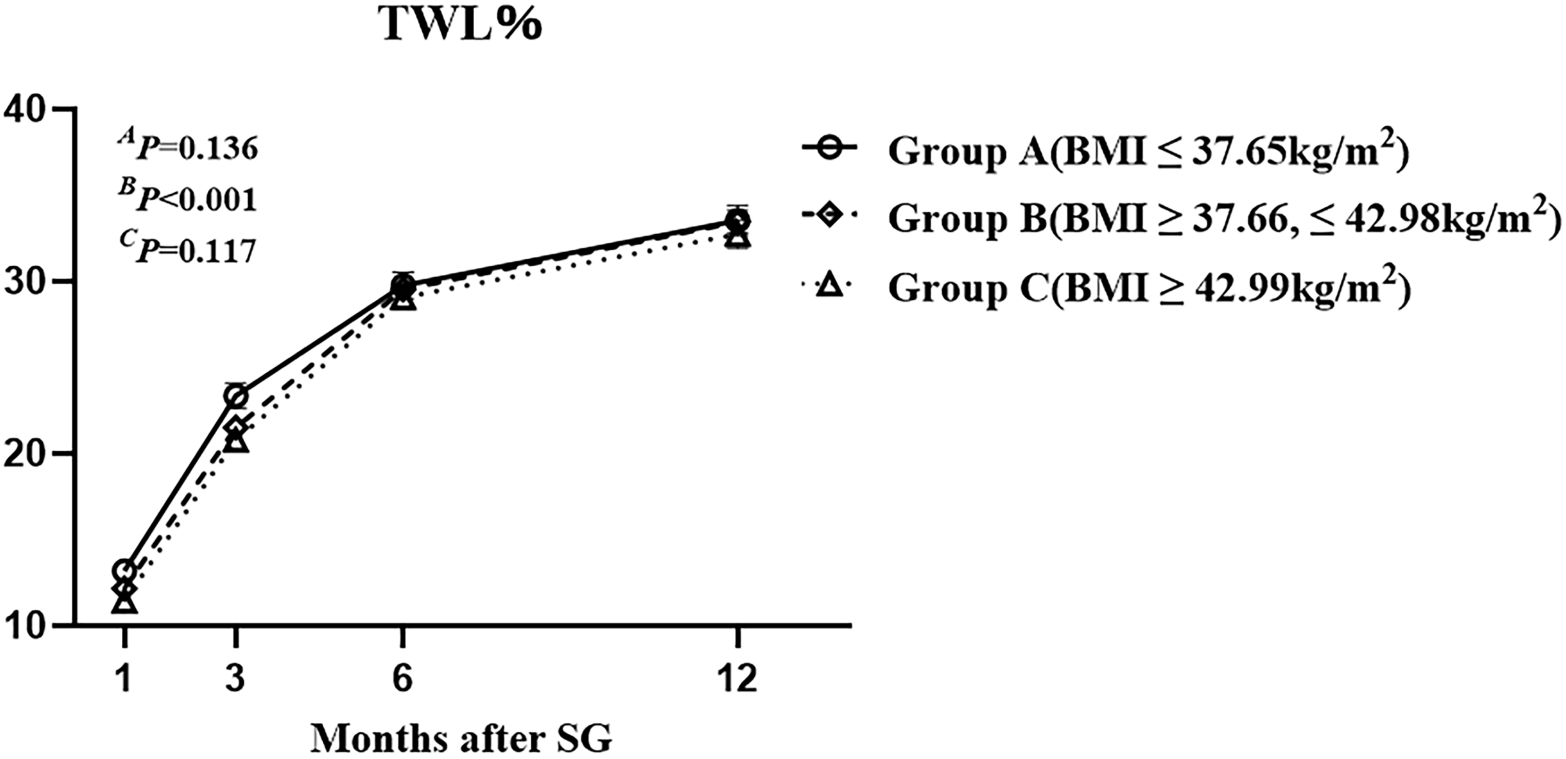
The total weight loss percentage of the three groups with different class of preoperative body mass index up to one year after sleeve gastrectomy. Total weight loss percentage was defined as weight loss/baseline weight *100%. ^A^ *P* by group, ^B^ *P* over time, and ^C^ *P* due to the interaction of the two factors.

The mean ( ± SEM) TWL% at 1, 3, 6, and 12 months after SG were 12.27 ± 0.21%, 21.90 ± 0.33%, 29.46 ± 0.40%, and 33.25 ± 0.46%, respectively. Overall, TWL% showed an upward trend within 1 year after SG. There were no significant differences among the three groups at 1,3,6, and 12 months after SG (**Figure 2**). The results indicated that the preoperative BMI did not correlate with postoperative weight loss effect.

### 3.4 Remission of irregular menstruation after SG

**Figure 3:**
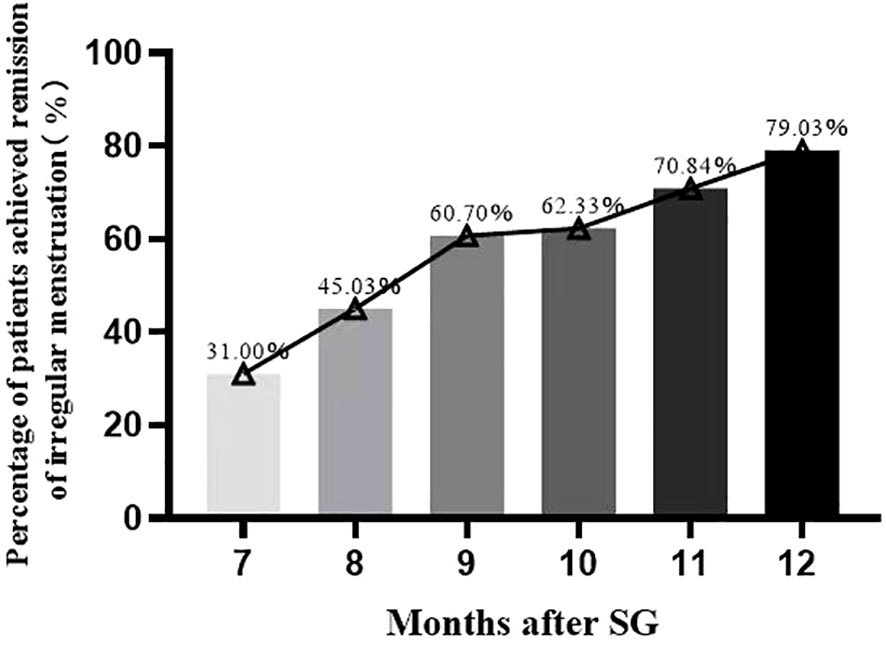
The percentage of patients achieved remission of irregular menstruation after sleeve gastrectomy.

After SG, 79.03% (181/229) and 20.97% (48/229) of the patients did and did not achieve remission of irregular menstruation in the one-year follow-up, respectively (**Figure 3**). It follows that SG achieved significant remission of irregular menstruation in patients with oligo-/anovulatory PCOS. Patients who achieved remission seven months after SG initiated menstruation within the first month after surgery. Regular menstrual cycles were initiated within the first and third months in 31.0% (71/229) and 60.7% (139/229) of patients, respectively. Interestingly, the patients who did not initiate regular menstruation within six months after SG continued to face this challenge for up to one year after SG. Therefore, the effect of SG on menstrual remission can be observed in the early postoperative period, and most patients with oligo-/anovulatory PCOS are sensitive to SG.

### 3.5 Correlation between preoperative BMI and remission of irregular menstruation after SG

**Figure 4:**
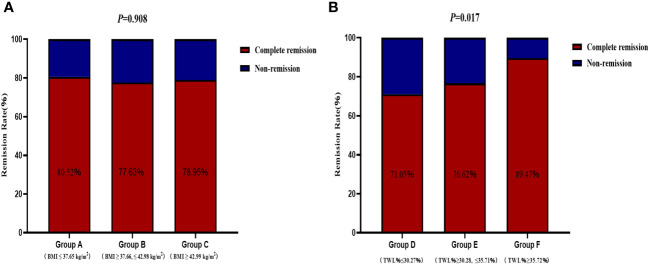
The correlations between remission of irregular menstruation and preoperative body mass index **(A)** and total weight lossp ercentage **(B)** at one year after sleeve gastrectomy.

**Figure 5:**
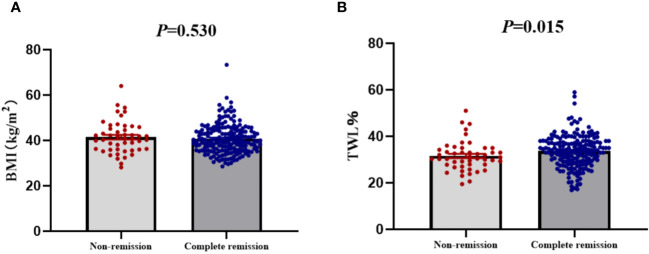
The compare of preoperative body mass index **(A)** and total weight loss percentage **(B)** between patients with complete remission and non-remission of irregular menstruation one year after sleeve gastrectomy.

The remission rates of irregular menstruation in groups A, B, and C were 80.52%, 77.63%, and 78.95%, respectively. No significant difference was detected among groups A, B, and C with respect to the remission rate (**Figure 4A**). Furthermore, there was no significant difference between patients with remission and without remission with respect to preoperative BMI (**Figure 5A**). Thus, there was no correlation between preoperative BMI and postoperative remission of irregular menstruation in patients with PCOS and obesity.

### 3.6 Correlation between TWL% and remission of irregular menstruation after SG

To further investigate the correlation between weight loss effect and remission of irregular menstruation, all participants were divided according to tertiles of TWL% at one year after SG (group D, ≤30.27%; group E >30.28%, ≤35.71%; group F >35.72%). The chi-square test results showed that there was a significant difference among groups D, E, and F with respect to remission rate (*P*<0.05) (**Figure 4B**). The result of univariate logistic regression analysis confirmed that TWL% at one year after SG [odds ratio (OR) 1.78, 95% confidence interval (CI) 1.18-2.69, *P* < 0.05] was a potential key factor that impacted remission of irregular menstruation after SG. And patients with remission of irregular menstruation had a higher TWL% at one year after SG compared to patients without remission (*P*<0.05) (**Figure 5B**).

## 4 Discussion

The principal finding of the current study is that SG is effective in the treatment of PCOS with a 79.03% remission of irregular menstruation one year after surgery. Furthermore, TWL%, rather than preoperative BMI, correlated with the remission after SG.

Irregular menstruation is a key concern in patients with PCOS even beyond infertility partially because of the increased risk of endometrial cancer (17). This study showed that 79.03% (181/229) of the patients achieved remission of irregular menstruation after SG, which is similar to that of a previous Chinese single-center study (78% remission rate) and higher than that of a study from Iran (66% remission rate) (18, 19). Another Chinese study by Meili Cai et al. (20) reported that 78.56% of patients achieved remission of irregular menstruation within six months after SG. The definition of remission in the study above required a consecutive three-month menstrual cycle (20). Limited by small sample sizes and inconsistency of remission standard of aforementioned studies, although the efficacy of BMS in the treatment of PCOS has been elucidated, it is underemphasized in present PCOS treatment guidelines. Our study, based on a prospective PCOS cohort with largest sample size, further confirmed that SG is effective in the treatment of irregular menstruation in Chinese patients with PCOS.

Considering that patients with PCOS and obesity have a more severe phenotype than those with PCOS and normal weight, including more severe menstrual irregularities and infertility (7, 21), it is reasonable to hypothesize that differences exist in menstrual remission after SG among patients with different preoperative body mass index (BMI) classes. To investigate this issue, we divided patients the into three groups (A, B, and C) according to tertiles of preoperative BMI. Surprisingly, our results indicated that no significant differences were detected among the three groups with respect to the remission rate. Obesity is the most common cause of insulin resistance and compensatory hyperinsulinemia, which are the primary pathological features of PCOS. Previous studies have demonstrated that insulin resistance plays a role in determining the degree of menstrual irregularity in patients with PCOS (22, 23). However, in the current study, for PCOS patients with an average BMI of 40.91 kg/m^2^, no significant differences were observed in HOMA-IR and AUC_insulin_ of OGTT values among the different BMI groups; therefore, it is reasonable for these patients to have a similar response to SG in the remission of irregular menstruation. This result further confirms that although obesity plays an important role in the occurrence and development of PCOS, the degree of obesity is not a key factor determining postoperative menstrual outcomes in patients undergoing SG.

Effective and sustained weight loss is the most fundamental change after SG. The follow-up results of this study also showed that patients with PCOS and obesity achieved an average TWL% of 33.25 ± 0.46% (with the range of 16.91% to 58.95%) 1 year after SG. This result was similar to that of a Chinese study (n=41) focusing on patients with PCOS and obesity, with an approximate TWL% of 33.38% (18). Furthermore, our study showed that the greater the TWL% achieved one year after SG, the greater the likelihood of remission of irregular menstruation. Hu et al. (18) concluded that endpoint BMI, rather than baseline BMI, is associated with the remission of irregular menstruation after SG. However, for patients with PCOS and obesity with an average BMI of 40.91 kg/m^2^ and a maximum BMI of 73.35kg/m^2^, it is unrealistic to simply pursue endpoint BMI without considering the difficulty of weight loss caused by baseline BMI. In our opinion, TWL%, which could reflect the magnitude of weight loss and concomitant metabolic changes, should be a better index for predicting remission of irregular menstruation after SG than the endpoint BMI.

BMS is superior to drug therapy for reducing hyperinsulinemia, and improving insulin sensitivity (24), both of which are optimal for treating PCOS. Improvement in insulin resistance, reduction of inflammatory markers, and loss of abdominal fat caused by sufficient weight loss can disrupt the vicious feedback cycle among obesity, inflammatory adipokines, and hyperinsulinemia, thereby improving menstrual irregularity (25–28). However, it is noteworthy that 60.7% of patients initiated regular menstruation within the third month after SG without sufficient weight loss, indicating the existence of a weight loss-independent effect of SG, which was similar to the mechanisms of diabetes remission after BMS.

To our knowledge, our study elucidated the correlation of postoperative menstrual remission with TWL% and preoperative BMI in patients with PCOS and obesity based on a multi-center cohort with the largest sample sizes. However, the participants were divided into groups based on tertiles of preoperative BMI and TWL% rather than international or Asian standards, which may limit the generalizability of the study results. Furthermore, a longer follow-up period is required to evaluate long-term effects and pregnancy outcomes.

The present study further confirmed that SG has a significant effect on menstruation remission in patients with obesity and PCOS. Preoperative BMI was not a decisive factor that correlated with remission; instead, TWL% emerged as a potential key factor.

## Data availability statement

The original contributions presented in the study are included in the article/supplementary material, further inquiries can be directed to the corresponding authors.

## Ethics statement

The studies involving humans were approved by the Medical Ethics Committee of Qilu Hospital of Shandong University. The studies were conducted in accordance with the local legislation and institutional requirements. The participants provided their written informed consent to participate in this study.

## Author contributions

YZ: Data curation, Formal analysis, Investigation, Methodology, Project administration, Software, Writing – original draft, Validation. SX: Data curation, Formal analysis, Writing – original draft. TL: Formal analysis, Writing – original draft, Methodology, Project administration, Software, Validation. JS: Formal analysis, Investigation, Methodology, Writing – original draft. TZ: Data curation, Methodology, Writing – original draft. SML: Supervision, Writing – review & editing. MZ: Supervision, Writing – review & editing. SZ: Conceptualization, Supervision, Writing – review & editing. XH: Writing – review & editing, Funding acquisition, Project administration, Validation. SZL: Writing – review & editing, Conceptualization, Funding acquisition, Project administration, Resources, Supervision, Validation, Visualization.
